# Autologous Dentin Graft after Impacted Mandibular Third Molar Extraction to Prevent Periodontal Pocket Formation—A Split-Mouth Pilot Study

**DOI:** 10.3390/ma15041431

**Published:** 2022-02-15

**Authors:** Giulia Mazzucchi, Marco Lollobrigida, Luca Lamazza, Giorgio Serafini, Dario Di Nardo, Luca Testarelli, Alberto De Biase

**Affiliations:** Department of Oral and Maxillofacial Sciences, Sapienza University of Rome, 00161 Rome, Italy; giulia.mazzucchi@uniroma1.it (G.M.); marco.lollobrigida@uniroma1.it (M.L.); luca.lamazza@uniroma1.it (L.L.); luca.testarelli@uniroma1.it (L.T.); alberto.debiase@uniroma1.it (A.D.B.)

**Keywords:** impacted lower third molar, autologous dentin graft, periodontal defect, third molar surgery

## Abstract

This preliminary study aims to evaluate the efficacy of an autologous dentin graft in preventing periodontal defects after impacted or semi-impacted lower third molars’ (M3) surgical extraction. For this purpose, radiographic and periodontal evaluation of post-extractive sockets were performed. Ten patients were enrolled in the study: twenty M3 extraction sockets were treated with a split-mouth modality. After tooth extraction, the experimental sites were filled with autologous dentin graft obtained by the extracted M3, while the control sites were filled with blood clot alone. Flaps were closed by first intention to ensure the stability of the wounds. Post-extractive sites were monitored at days 15, 90 and 180. The healing was not affected by any complications associated with the use of the autologous dentine graft in all cases. The measurements recorded at six months showed a reduction of the probing pocket depth distal to the second lower molar (M2) at both surgical sites, with a greater reduction observed at the experimental sites. Radiographic evaluation also showed a greater amount of bone gain at the grafted sites compared to the control sites. The results of this preliminary study suggest that autologous dentin grafts can be useful in preventing the formation of periodontal defects distal to M2 after M3 surgical extraction.

## 1. Introduction

Surgical extraction of impacted lower third molars (M3) is one of the most common procedures in oral surgery. M3s can be found totally or semi-impacted in the bone, most frequently in horizontal or mesioangular position [1]. Semi-impacted M3s exposed to the oral environment or totally impacted M3s in direct contact to the adjacent second molar (M2) are more prone to soft tissue inflammation, acute or chronic infection, pain, caries of M2, interdental bone loss and development of a periodontal socket distal to M2 [2,3,4].

These conditions may suggest third molar extraction in order to avoid future complications, even for young patients. Significantly, it has been reported that, in patients older than 26 years with mesioangular or horizontal impaction of M3 and preexisting periodontal defects, the postoperative periodontal healing of quite the 50% of M2 can be compromised, with a postoperative probing depth > 7 mm in 43.3% of these subjects [3,5,6,7].

In some cases, the extractive procedures themselves, due to the surgical technique or postoperative complications (wound infection, collapse of the flap and foreign body reaction), can lead to complicated and long healing with the formation of dehiscence associated to a persistent or developing periodontal pocket on the distal aspect of M2 [8].

Different bone substitutes (allografts, xenografts, autografts) have been proposed as post-extractive graft materials to prevent periodontal defects distal to M2. Among the graft materials, only the autologous bone is characterized by osteoinductive, osteoconductive and osteogenic properties, and for this reason it is widely considered as a gold standard [9,10,11]. However, a standardized regenerative approach has not been established yet.

In this scenario, the use of extracted teeth has been introduced in recent years as autologous grafts alternatively to conventional bone substitutes [12,13]. The use of dentin in the form of a demineralized dentin matrix (DDM) has been proposed due to its biological and structural similarities with alveolar bone [14,15,16,17]. Moreover, teeth and bone have the same embryological origin, since they both derive from the cells of the neural crest [18]. Alveolar bone is composed of 65% inorganic content and 25% of organic content; in turn, dentin has an inorganic content of about 70%–75% and an organic content of about 20%. At least 90% of the organic content of dentin is represented by type I collagen, which plays an important role in bone formation and mineralization, while the remaining portion is composed of other non-collagenic proteins such as osteocalcin, osteonectin and phosphoprotein [19]. Dentin also contains bone morphogenetic proteins (BMPs), which promote the differentiation of mesenchymal stem cells into chondrocytes, and consequently stimulate bone formation [20]. Different studies have demonstrated the osteoinductive capacity of DDM, highlighting an osteoinductive action of dentin-derived BMPs similar to that observed for bone matrix-derived BMPs [20,21].

Considering the osteoinductive and osteoconductive properties of DDM, different grinding devices have been introduced to obtain particulate graft from extracted teeth. Autologous dentine graft can be then directly inserted into the post-extractive sites as a ridge preservation technique, or at sites where the bone regeneration is needed [22]. Notwithstanding, there are still few studies in the literature that have evaluated the clinical outcomes of dentin grafts [12,16,18,21,22].

The aim of this study was to evaluate periodontal and radiological outcomes of the use of an autologous dentin graft in preventing periodontal defects on the distal aspect of M2 after impacted M3 surgical extraction. The study hypothesis was that a post-extractive dentin graft can reduce the probing pocket depth (PPD) (primary outcome) and improve radiographical bone gain (secondary outcome) at the distal aspect of M2.

## 2. Materials and Methods

This split-mouth pilot study was performed in a single center at the Oral Surgery Unit of the Department of Oral and Maxillofacial Sciences, “Sapienza” University of Rome, Italy.

Eligibility criteria included:

- Subjects requiring the extraction of partially or totally impacted third molars, with horizontal or mesioangular position, due to recurrent pericoronitis, destructive caries of the third molar, destructive caries of the second molar not otherwise treatable except after extraction of the third molar, periodontal defects distal to the second molar;
- Radiographic or clinical evidence of bone loss between the distal aspect of M2 and the adjacent M3. The assignment to the experimental or control group was carried out by a software-controlled randomization procedure;
- Patients ≥ 18 years of age;
- Presence of mandibular second molar adjacent to the third molar to be extracted;
- Willingness and ability to give written informed consent;
- Non-smoking or light smoking patients (≤10 cigarettes per day);
- Willingness to return for follow-up examinations;
- Good general health.

Moreover, the absence of severe inflammation at the experimental sites was considered mandatory to avoid complicated or delayed healing.

Subjects presenting any of the following exclusion criteria were not admitted to the study:

- Uncontrolled systemic diseases/conditions (e.g., pregnancy or lactation, drug or chemical reagents hypersensitivity, diabetes, metabolic bone diseases, history of malignancy);
- Heavy smokers (>11 cigarettes/day);
- Long-term steroidal drug therapy;
- Unwillingness to return for follow-up appointments.

**Figure 1:**
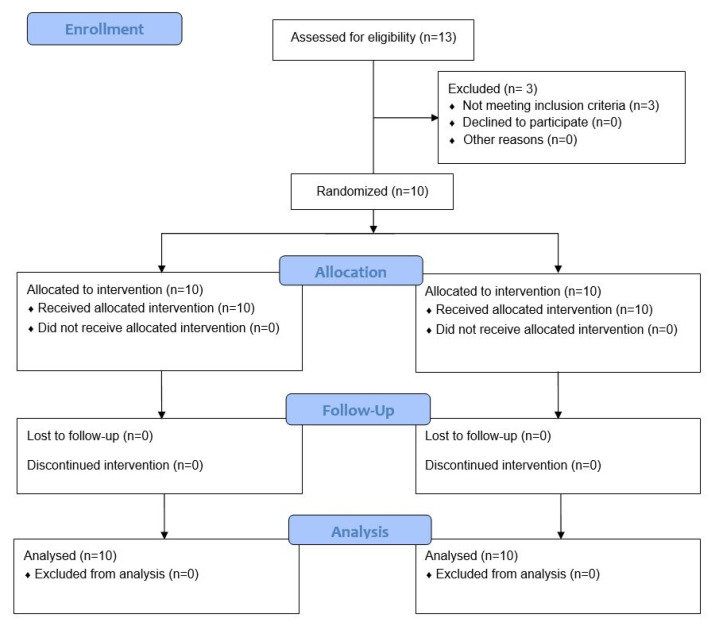
CONSORT flow diagram. Enrolled subjects participated in both study groups (split mouth) with a 3-week washout.

Thirteen subjects were assessed for eligibility. Of these, ten patients (6 women and 4 men; ≥18 years; age range 18–44; mean age 32.7 ± 9.8) respected the eligibility criteria and were enrolled in the study (Figure 1).

A split-mouth allocation of post-extraction sockets to the experimental or control group was carried out for each patient by a software-controlled randomization procedure. Allocation was made before starting interventions but was revealed to the surgeon only after tooth extraction in order to eliminate any sources of procedural bias. Experimental sites were treated with autologous dentine graft while control sites were only filled by blood clot. Patients were regularly followed-up for 6 months. Blinding procedures could not be performed due to the nature of the intervention.

This study followed the principles of the Declaration of Helsinki related to the work of research involving human species and received the approval of Policlinico Umberto I Ethics Committee (Ref. no. 5456/2019). Patients were enrolled in the study after signing a written informed consent.

After initial examination, an oral hygiene session was established for each patient. Before surgery, periapical radiographs of the sites (M2 and M3) were taken, and periodontal indexes were recorded. Periapical radiographs were taken using phosphor plates. Due to the difficulty in using Rinn holders in the posterior mandible, a customized holder was then adopted to keep the plate perpendicular to the X-rays and parallel to the long axis of the teeth [10]. Plaque index (PI), bleeding on probing (BOP), gingival index (GI), clinical attachment level (CAL) and PPD were recorded at mesiobuccal, midbuccal, distobuccal, middistal, distolingual, midlingual and mesiolingual sites of each M2, with a PCP-15-UNC probe (Hu-Friedy, Leimen, Germany) [23,24,25].

Clinical and radiographic evaluations were carried out by an expert and blinded calibrated operator (G.M.), with a manual pressure of about 25 g, previously tested on a digital load cell.

**Figure 2:**
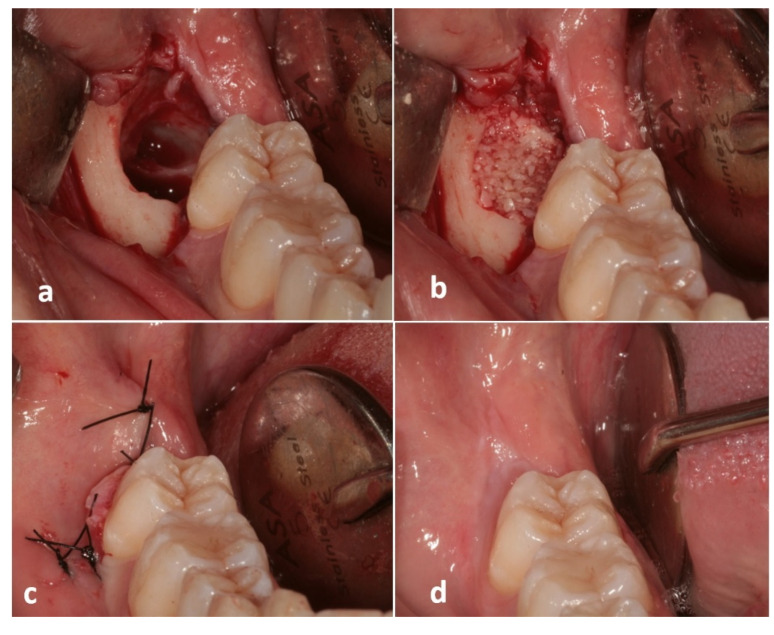
Clinical images showing the treatment sequence at an experimental site: (**a**) Post-extractive third molar socket; (**b**) Autogenous dentin graft filling the socket; (**c**) Flap suture with primary closure; (**d**) Soft tissue healing at 180 days after surgery.

**Figure 3:**
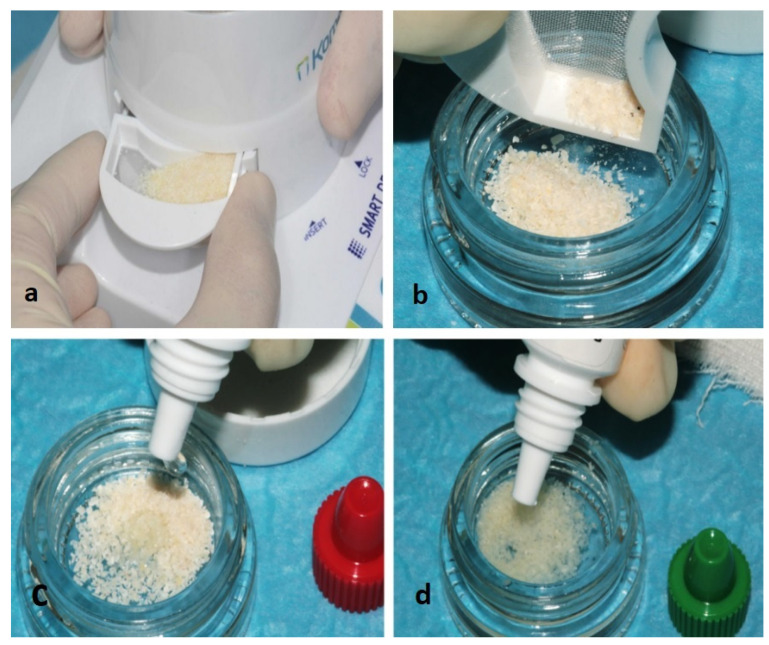
Dentin graft preparation: (**a**) Particulate was obtained with a diameter of 300 to 1200 µm; (**b**) The particulate was inserted in a sterile dappen, (**c**) rinsed with a 0.5 M NaOH and 20% ethanol solution for 5 min and then (**d**) rinsed two times for 3 min with a phosphate-buffered saline solution.

Surgical interventions (Figure 2) were all performed by the same skilled clinician (M.L.). Under local anesthesia (troncular anesthesia with 3% mepivacaine without vasoconstrictor and local infiltration of 2% mepivacaine with 1:100,000 epinephrine) a full-thickness flap was elevated; ostectomy and odontotomy were performed with a multiblade tungsten carbide cutter and tooth fragments mobilized by elevators. In order to minimize the loss of alveolar bone, a minimally invasive protocol was adopted [26]. Any granulation tissue was carefully curetted. The root surface distal to the second molar were also curetted with Gracey curettes. At the experimental sites, following the manufacturer’s instructions, M3’s fragments were cleaned with a high-speed tungsten carbide bur, dried and grinded for 3 s by the use of a dedicated device (Smart Dentin Grinder™, KometaBio, Fort Lee, NJ, USA), producing particles of 300–1200 μm. The granules were immersed into a 0.5 M NaOH and 20% ethanol solution for 10 min to dissolve organic remains and then rinsed twice (3 min each) with phosphate-buffered saline solution (Figure 3) [27]. The use of the tungsten carbide bur involved the elimination of a thin layer of enamel; however, most of the enamel was preserved and grinded with the rest of the tooth. Enamel mainly acts as an inert mineralized component, supporting the graft and integrating into the bone, while the bioactive component of the graft is represented by dentin due to the presence of BMP; for this reason, the particulate is generally referred to as “dentin graft”.

At the experimental sites, the post-extractive socket was filled with the grinded dentin, gently condensed, while control sites were only filled by the blood clot. No barrier membranes were used. In case of total impaction, the flaps were repositioned and stabilized with a 4.0 nylon suture with a primary closure. In case of partial impaction, a transposed flap was adopted to avoid flap tension and obtain a primary closure [28]. Antibiotics were administered for 6 days (1 tablet of amoxicillin 875 mg + clavulanic acid 125 mg every 12 h) associated with local antiseptics (chlorhexidine digluconate 0.20% mouth rinses twice a day for 14 days). Non-steroidal anti-inflammatory analgesics (nimesulide 100 mg) were also prescribed (100 mg every 8 h only in case of pain).

**Figure 4:**
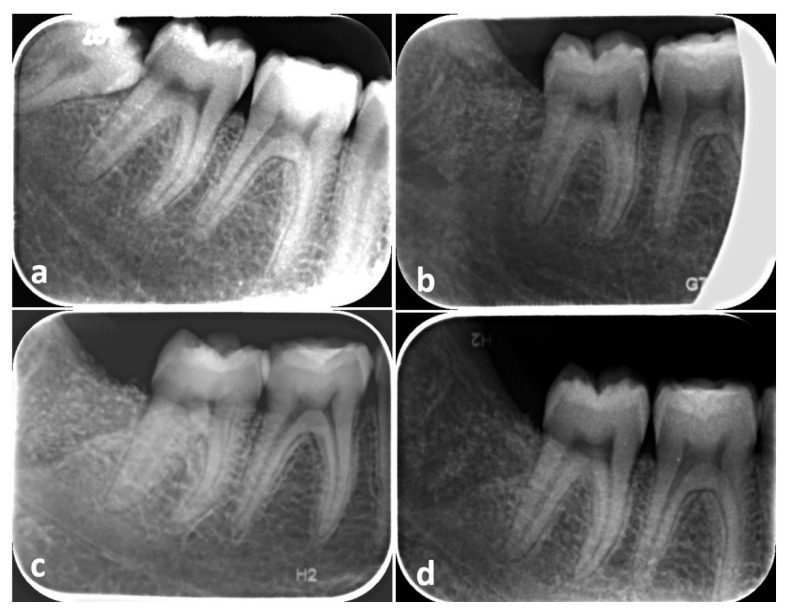
Radiographic images showing the treatment sequence at an experimental site: (**a**) Presurgical periapical radiograph; (**b**) 15 days, (**c**) 90 days and (**d**) 180 days after socket grafting.

At day 15, sutures were removed, and periapical radiographs were taken in order to control post-extractive conditions. At days 90 and 180, new periapical radiographs and periodontal indexes were re-evaluated for both sites (Figure 4). A period of 6 months of follow-up was deemed sufficient, as many histological randomized controlled trials showed that 20–21 weeks is considered the sufficient time to obtain evidence of bone regeneration [29,30,31,32,33,34].

An operator (G.M.) who was not informed of the treatment undergone by the individual patients measured preoperative and postoperative bone level distal to M2 on periapical radiographs taken at different follow-up times by the software AutoCAD 2017 (Autodesk, San Rafael, CA, USA), then validated by Invivo software (Anatomage Inc., San Jose, CA, USA) and 3D Endo Software (Dentsply Sirona, Charlotte, NC, USA). The radiographic bone level was measured at T1 and T180 as the distance between the distal point of a line passing through the cemento-enamel junction of M2 (mesial and distal projection of M2’s CEJ) and the bone peak distal to M2, at the bottom of the bony defect, as described in a previous study [35]. Bone gain (BG) was considered as the difference between bone level at T1 and T180.

Due to test differences in terms of PPD at T1, T90 and T180 and differences in terms of CEJ-bone peak at T1, T180 and BG between the experimental and the control sides, we performed the Wilcoxon signed rank tests as appropriate for paired data with nonparametric distribution. The analysis was performed using SPSS software version 13.0 for Windows (SPSS Inc., Chicago, IL, USA) and statistical significance level was set at *p* ≤ 0.05.

## 3. Results

Each of the ten patients (6 women and 4 men; ≥18 years; age range 18–44; mean age 32.7 ± 9.8) underwent the extraction of both mandibular M3s. Three subjects were light smokers (<10 cigarettes per day). All the procedures were successfully carried out and no patients showed complications like pain or swelling of the post-extractive sites.

**Figure 5:**
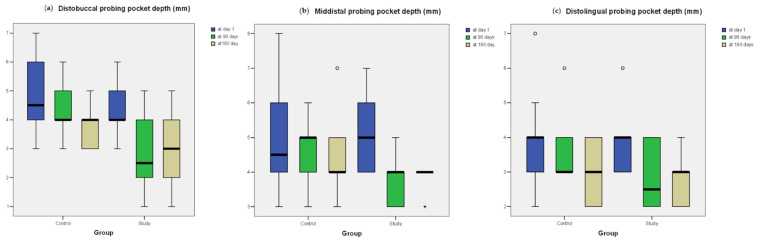
Box plots of probing pocket depth (PPD, mm) of the distal aspect of the experimental site and the control site at different follow-up intervals. (**a**) distobuccal PPD; (**b**) middistal PPD; (**c**) distolingual PPD. Outlier values at a distance from the median within 1.5 and 3.0 times the interquartile range (IQR) are plotted as circles. Extreme values at a greater distance from the median are plotted as asterisks.

**Figure 6:**
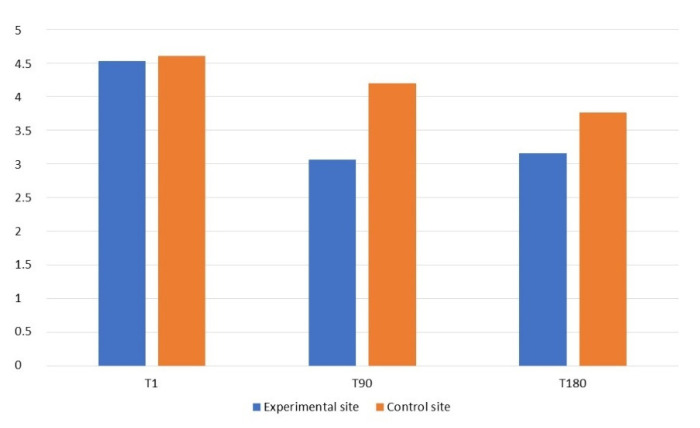
Graph of cumulative (distobuccal, middistal, distolingual) probing pocket depth (PPD, mm) of the distal aspect of the experimental site and the control site at different follow-up intervals.

The data of the periodontal examination are reported in Table 1 and Figure 5 and Figure 6.

The comparison of the probing pocket depth mean values (mean PPD) of M2′s distal aspect (distolingual, middistal, distobuccal) at times T1, T90 and T180 showed a positive trend in all parameters, recorded both at the experimental and the control sites (Table 1). A decrease of the PPD values was observed at both experimental and control sites, but with a greater reduction at the grafted sites. PPDs at the experimental sites decreased from an average of 4.53 ± 1.13 mm at T1 to an average of 3.16 ± 0.98 mm at T180, with a mean difference of 1.37 mm, while the PPDs at the control sites decreased from an average of 4.6 ± 1.67 mm at T1 to an average of 3.76 ± 1.04 at T180, with a mean difference of 0.84 mm. Specifically, the differences in terms of PPD at T1 and T180 were not significant on the distobuccal (*p =* 0.368 and 0.065, respectively), middistal (*p =* 0.944 and 0.096, respectively) and distolingual sites (*p =* 1.000 and 0.414, respectively). However, the differences were statistically significant at T90 on each of the three sites (distobuccal: *p =* 0.014; middistal: *p =* 0.014; distolingual: *p* = 0.033).

**Figure 7:**
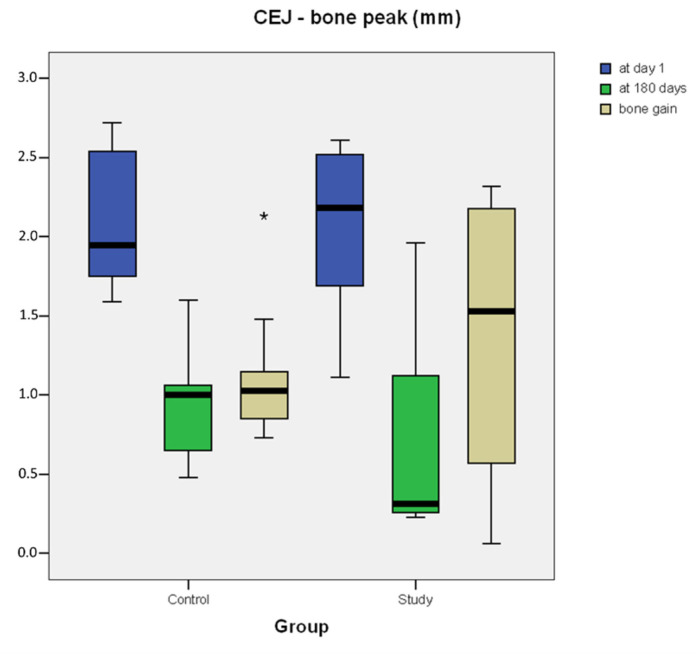
Box plot of radiographic measurements from distal point of M2’s CEJ to bone peak (mm) at T1 and T180. Bone gain (BG). Outlier values at a distance from the median within 1.5 and 3.0 times the interquartile range (IQR) are plotted as circles. Extreme values at a greater distance from the median are plotted as asterisks.

Radiographic analysis is reported in Table 2 and Figure 7. The comparison of the periapical intraoral radiographs taken at T1 and T180 showed that bone peaks distal to the second molar of both sites at the end of the follow-up period gained a more coronal position compared to preoperative values, showing a different trend in the various phases of the study protocol. Measurements show that, in the experimental sites, the bone peak distal to M2 was on average higher following the graft procedure, with a mean value of BG of 1.403 ± 0.87 mm after six months. Moreover, at the control sites filled with blood clot alone, the bone peak healing reaches a more coronal position than preoperative situation, with a mean value of BG of 1.125 ± 0.41 mm at T180 (Table 2). However, no statistically significant differences were detected when comparing the CEJ–bone peak value at T1 (*p =* 0.646), T180 (*p =* 0.169) and BG (*p =* 0.508).

## 4. Discussion

Mandibular second molars adjacent to impacted or semi-impacted third molars are more prone to develop periodontal pathologies [36]. Several studies have shown that postoperative periodontal healing of quite half of M2 seems to be compromised with a postoperative probing pocket depth > 7 mm in 43.3% of the cases, even 4 years after M3′s extraction [3,5,6,7,37]. To decrease the risk of clinical attachment loss on the distal aspect of M2, different approaches have been proposed, including specific flap designs [28], the use of bone substitutes and barrier membranes. The present pilot study highlighted an improvement in periodontal indexes and radiographic bone gain distal to M2 in post-extractive sites treated with autologous dentin graft. The results are in accordance with the findings of systematic reviews and meta-analyses which highlighted the importance of regenerative treatment to prevent periodontal defects distal to M2 after M3 extraction [3,7]. In particular, it has been observed that the use of conventional bone substitutes, compared to spontaneous wound healing, are effective in gaining clinical attachment level and alveolar bone level, reducing PPD without an augmented risk of postoperative complications.

In the literature, there are no randomized clinical studies that have evaluated the efficacy of autologous dentin grafts in the prevention of periodontal defects. Consequently, the results of this study can only be compared with those of other split-mouth studies testing other graft materials. A randomized controlled clinical study by Sammartino et al. [38] on the post-extractive sites of 93 mandibular third molars, with a 72-months follow-up, demonstrated that the combined use of bovine mineral bone and a collagen membrane can achieve an improvement in terms of PPD and CAL of the distal site of M2, as compared to the use of bovine bone alone, and that both these treatment modalities have advantages compared to spontaneous healing. Similar results were reported by Hassan et al. [39], who evaluated the use of bovine bone combined with a resorbable membrane compared to spontaneous healing after M3’s surgical extractions. At the end of the 12-months follow-up, the PPD was 3.1 ± 0.4 mm vs. 7.5 ± 0.7 mm (baseline) for grafted sites and 4.8 ± 0.5 mm vs. 7.8 ± 0.8 mm for the nongrafted sites, with a bone gain of 3.59 mm for grafted sites against 1.20 mm for nongrafted sites. Leventis et al. [40], in a case series with a mean follow-up of 1.5 years, investigated the combined use of beta-tricalcium phosphate and calcium sulfate in post-extractive sockets of M3 in patients with preexisting periodontal pockets distal to M2. At the end of the follow-up period, the authors found a mean PPD of 2.00 ± 0.71 mm and a radiographic bone gain of 6.07 ± 0.28 mm distal to M2.

Although the potential of membranes to promote healing, as well as their barrier effect to prevent epithelial migration into the wound is well known [3,41], in this study no membranes were used. Since a primary intention closure was obtained in all cases, the authors decided to use the autologous dentine graft alone, exploiting its scaffold capacity, osteoinductive and osteoconductive properties, thus avoiding possible complications deriving from the use of resorbable membranes.

The authors are aware of the intrinsic limitations of the study presented. First, the small sample size represents only a convenient sample, since there are no prospective reports in the literature that evaluated the use of dentin graft in preventing pocket formation after third molar extraction. In this regard, the absence of a statistical significance for most comparisons may be the results of the small sample size rather than the absence of a beneficial effect of the grafting procedure. Other limitations are represented by the medium-term follow-up, which may discard long-term incidence of periodontal pathology of second molars, and the lack of a histological evaluation of the grafted site to appreciate the composition of the newly formed bone. Notwithstanding, the results of this study are encouraging and advocate for further investigation. Compared to other osteoconductive bone substitutes, dentin grafts present further advantages, since they are costless for patients, completely biocompatible and osteoinductive. One limitation could be the limited amount of particulate that can be obtained from extracted teeth, depending on their dimensions and on the surgical technique (odontotomy). However, a solution could be the concomitant extraction and grinding of the antagonist upper third molar.

## 5. Conclusions

The results of this study suggest that the use of an autologous dentin graft after the surgical removal of mandibular third molars may be useful in preventing postoperative periodontal pockets distal to the second molar.

Considering the limitations of this study, further research with greater sample size, histological evaluation and longer follow-up periods is mandatory to confirm these preliminary results. If the data obtained in this study will be confirmed, autologous dentin grafts could become a reliable material in the treatment of post-extractive sites to prevent pocket formation after third molar surgery.

## Figures and Tables

**Table 1:** Patient’s characteristics: gender, age, light-smoker (<10 cigarettes/day) or nonsmoker, type of inclusion; probing pocket depth (PPD, mm) of the distal aspect of the experimental site and the control site at different follow-up intervals; mean values of PPD. PPDs > 3 mm are reported in bold.

Case	Gender	Age	Smoker	Type of Inclusion			Experimental Site PPD			Control Site PPD		
				Esperimental Site	Control Site		T1	T90	T180	T1	T90	T180
1	F	29	No	Semi–Impacted	Semi–Impacted	Distobuccal	4	4	3	4	4	4
						Middistal	4	3	3	4	4	4
						Distolingual	3	3	3	2	3	2
2	M	18	No	Semi–Impacted	Semi–Impacted	Distobuccal	4	2	2	3	4	4
						Middistal	6	4	3	3	4	4
						Distolingual	4	2	2	3	3	2
3	F	44	Light	Semi–Impacted	Semi–Impacted	Distobuccal	6	2	3	5	3	3
						Middistal	6	4	4	8	5	4
						Distolingual	6	4	3	4	3	3
4	M	37	No	Sem–Impacted	Semi–Impacted	Distobuccal	5	3	2	3	5	3
						Middistal	7	4	4	6	5	4
						Distolingual	4	2	2	2	3	2
5	M	26	Light	Semi–Impacted	Semi–Impacted	Distobuccal	3	1	1	6	4	4
						Middistal	5	3	4	3	4	3
						Distolingual	3	2	4	4	4	4
6	F	39	No	Semi–Impacted	Semi–Impacted	Distobuccal	4	3	3	7	6	5
						Middistal	6	4	4	5	5	7
						Distolingual	4	4	3	4	4	4
7	M	31	Light	Semi–Impacted	Semi–Impacted	Distobuccal	3	1	1	6	5	4
						Middistal	5	3	4	8	5	5
						Distolingual	3	2	4	3	3	3
8	F	29	No	Semi–Impacted	Sem–Impacted	Distobuccal	5	2	4	4	3	3
						Middistal	4	3	4	4	3	4
						Distolingual	4	2	2	4	3	3
9	F	33	No	Semi–Impacted	Impacted	Distobuccal	4	4	5	7	6	5
						Middistal	4	4	4	6	6	4
						Distolingual	4	4	3	7	6	4
10	F	41	No	Semi–Impacted	Semi–Impacted	Distobuccal	6	5	4	4	4	3
						Middistal	5	5	4	4	5	5
						Distolingual	3	3	3	5	4	4
						Mean	4.53 ± 1.13	3.06 ± 1.08	3.16 ± 0.98	4.6 ± 1.67	4.2 ± 1.03	3.76 ± 1.04

**Table 2:** Radiographic measurements from distal point of M2’s CEJ to bone peak (mm) at T1 and T180. Bone gain (BG). Mean values of distance in mm.

Case	Experimental Site			Control Site		
	CEJ–Bone Peak (mm)			CEJ–Bone Peak (mm)		
	T1	T180	BG	T1	T180	BG
1	1.11	0.24	0.87	1.59	0.59	1
2	2.61	0.29	2.32	1.59	0.65	0.94
3	1.24	0.23	1.01	1.78	0.98	0.8
4	2.52	0.34	2.18	2.38	1.23	1.15
5	1.70	1.13	0.57	2.61	0.48	2.13
6	2.44	0.39	2.05	2.72	1.6	1.12
7	2.02	1.96	0.06	2.54	1.06	1.48
8	1.69	1.12	0.57	1.87	1.02	0.85
9	2.35	0.26	2.09	2.02	0.97	1.05
10	2.58	0.27	2.31	1.75	1.02	0.73
Mean	2.026 ± 0.56	0.623 ± 0.58	1.403 ± 0.87	2.085 ± 0.43	0.96 ± 0.33	1.125 ± 0.41
Median (interquartile range)	2.185 (0.8075)	0.315 (0.675)	1.53 (1.1525)	1.945 (0.7425)	1 (0.32)	1.025 (0.27)

## Data Availability

The data presented in this study are available on request from the corresponding author.
